# Novel Modular Walking Orthosis (MOWA) for Powerful Correction of Gait Deviations in Subjects with a Neurological Disease

**DOI:** 10.3390/children11010030

**Published:** 2023-12-26

**Authors:** Jan-Hagen Schröder, Gion A. Barandun, Pascal Leimer, Rafael Morand, Beat Göpfert, Erich Rutz

**Affiliations:** 1 MOWA Healthcare AG, 4500 Solothurn, Switzerland; jan-hagen.schroeder@mowa.com; 2 IWK Institute for Materials Technology and Plastics Processing, Eastern Switzerland University of Applied Sciences, 8640 Rapperswil, Switzerland; gion.barandun@ost.ch; 3 Switzerland Innovation Park Biel/Bienne, 2503 Biel, Switzerland; leimer@pm.me; 4 Biomedical Engineering Lab, Institute for Human Centered Engineering, Bern University of Applied Sciences, 3008 Bern, Switzerland; rafael.morand@students.unibe.ch; 5 Department Biomedical Engineering (DBE), University of Basel, 4123 Allschwil, Switzerland; beat.goepfert@unibas.ch; 6 Laboratory for Movement Analysis, University of Basel Children’s Hospital (UKBB), 4056 Basel, Switzerland; 7 The Royal Children’s Hospital, Melbourne, VIC 3052, Australia; 8 Bob Dickens Chair Paediatric Orthopaedic Surgery, The University of Melbourne, Melbourne, VIC 3010, Australia; 9 Murdoch Children’s Research Institute, Melbourne, VIC 3052, Australia; 10 Medical Faculty, University of Basel, 4001 Basel, Switzerland

**Keywords:** equinus deformity, foot drop, cerebral palsy, hemiplegia, digital manufacturing, 3D printing, lower leg orthosis, artificial intelligence, MOWA (modular walking orthosis)

## Abstract

This article introduces a novel concept where advanced technologies have been leveraged to produce a modular walking orthosis (MOWA) within a completely digital process chain. All processes of this new supply chain are described step-by-step. The prescription and treatment of lower leg orthoses for individuals with paralysis or muscle weakness, particularly cerebral palsy (CP), are complex. A single case study indicates successful treatment with this new orthosis (MOWA). From the authors’ perspective, this innovative fitting concept is promising and will contribute to creating more efficient care within a multidisciplinary team.

## 1. Introduction

This article introduces an innovative integrated concept to address this challenge, wherein a modular orthosis (MOWA) is produced using advanced technologies within a completely digital process chain. Fitting and manufacturing orthoses for individuals with paralysis, particularly those with cerebral palsy (CP), present inherent complexities. Equinus deformity is the most common problem in children with CP. Conservative management includes physical therapy, the injection of botulinum toxin, or orthotics (ankle–foot orthosis = AFO). In some cases, fitting a standard AFO might be challenging. This process takes into account relevant parameters specific to each case right from the outset. The authors believe that these new treatment concepts hold promise and will significantly improve the efficiency of supplying patients with appropriate devices, particularly when applied in a multi-disciplinary team.

The adaptation of a need-based orthopedic technical aid requires a systematic approach [1]. In lower leg prosthetics, this requirement has been met for years, and a great deal of technical and financial effort has been invested, both in scientific research and industry. The biomechanical properties of prosthetic fittings have been defined more and more clearly in recent decades, enabling precise tailoring to the respective amputation level, including considerations of weight, activity level, and areas of application of the prosthesis. On the other hand, orthotic care faces more complex challenges.

In contrast to amputation, in lower limb orthotics, the extremity to be supported is still present and may only fulfil the function of locomotion in a reduced manner or be unable to provide support altogether. This is often due to complete or incomplete paralysis, combined with spasticity and/or joint contractures. From a medical standpoint, it is crucial to differentiate between flaccid and spastic limbs. This differentiation typically has implications for the gait cycle, affecting the following aspects:

- Problems in the stance phase (typically caused by spasticity with increased activity of the plantar flexors);
- Problems in the swing phase (usually resulting from weakness or paralysis of the dorsal extensors in the ankle joint, resulting in drop foot).

The goal of any orthosis for a patient with neurologically conditioned walking disorders should be to improve or even restore the physiological ability to walk and stand (normal gait). Therefore, it is essential to distinguish between stance and swing phase problems as this differentiation significantly impacts the design and functionality of the orthosis. To make accurate decisions, conducting an adequate anamnesis and diagnosis is crucial. The aim of this publication is to describe all the steps of this new supply chain.

## 2. Workflow and Supply Chain

### 2.1. Medical History

To precisely determine the required function of an orthosis, an extensive anamnesis and a detailed physical examination of the patient by the care team are indispensable. The study of the following aspects is particularly important:

- Motor failures;
- Residual function of the musculature;
- Degrees of freedom of the joints (range of motion);
- Axis deviations of the knee or ankle joint.

In addition, it is essential to record the specific characteristics, needs, and abilities of the patient. For instance, in the case of a subject with spastic hemiplegia, the ability to tighten and close the lower leg orthosis using only one hand is critical. Such special requirements provide crucial information for the future design of the orthosis.

### 2.2. Diagnosis

The gold standard for diagnosing a gait disorder is a three-dimensional instrumented gait analysis (3DGA). This method allows for the precise measurement of joint angles (kinematics) in all three planes and the calculation of associated forces (inverse dynamics). However, these systems are often not available and do not provide immediate suggestions for the design of the orthosis.

### 2.3. Problem

Currently, orthoses in a wide variety of implementations exist on the market, offering different levels of support depending on the design and the material. However, there is currently no consensus based on scientific data regarding the selection of an orthotic design suitable for a specific indication in the orthopedic technical field.

From the experience of the authors, this often leads to patients being treated following a trial-and-error principle. Patients may receive a standard orthosis that lacks individual fit and comfort, or they might be provided with a custom-made orthosis using a plaster cast model. The optional integration of joints further allows for tailoring aid to the patients’ individual needs.

### 2.4. MOWA (Modular Walking Orthosis)—A New, Systematic Fitting System for the Digital

#### Creation of Effective Custom Orthoses

As mentioned before, numerous factors need to be considered when designing an orthosis. According to the experience of the authors, the future of orthotics lies in the greatest possible integration of state-of-the-art production, information, and communication technology [2]. In this context, the term ‘Industry 4.0’ is often used. Such a concept would promote the collaboration of interdisciplinary care teams and facilitate the creation of optimal orthotic aids for patients of all ages with neurologically related musculoskeletal disorders.

**Figure 1:**
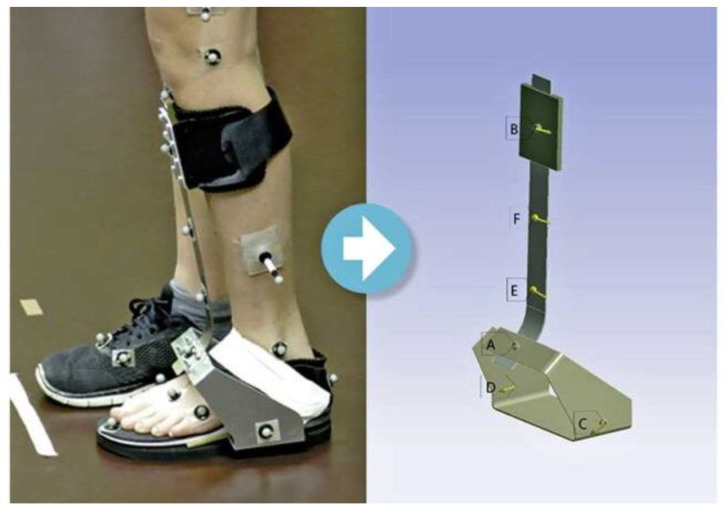
(**Left**): Prototype orthosis with standardized marker set up during gait by 3D gait analysis. (**Right**): Transfer of deformations into simulation software (finite element modelling, FEM) for the calculation of forces and torque in the orthosis at different points A–F.

A digital process chain spans from assessment and planning to the creation of the orthosis to close follow-up control and complete documentation. Such a concept is made possible by the use of 3D gait analysis (3DGA) and simulation technology, which allows for cause-and-effect relationships [3,4]. In combination with artificial intelligence, the design of the orthosis can then be tailored to the individual needs of the subject (Figure 1).

A new system for lower limb orthosis fitting and manufacturing based on this technology is the MOWA concept. MOWA stands for modular walking and was developed in Switzerland over the last five years. Currently, it is being introduced throughout Europe and other countries worldwide. The system relies on a large dataset from gait analyses (3DGA), forming the basis for training the artificial intelligence behind this innovative system. This enables a reliable, standardized classification of the patients’ deficits and, consequently, the creation and fitting of optimal orthoses. The data used come from customers of Orthopunkt AG, an orthotic care center, and MOWA Healthcare AG, who have provided their consent for data analysis. By evaluating the data collected by the technician on the respective subject, the system immediately generates a concrete recommendation regarding the optimal orthosis configuration. The selected, dynamic carbon components of the MOWA orthosis are then combined with individual, customized 3D-printed parts. Thanks to the glue-free connection of all the components, the orthosis becomes modular and can be adjusted whenever the patient’s gait pattern changes or a child grows. The system was developed in cooperation with the following Swiss companies:

- Orthopunkt AG—Centre for Technical Orthopedics, Solothurn;
- MOWA Healthcare AG, Solothurn;
- Composites Busch SA, Porrentruy.

This research work was supported by Innosuisse, Swiss Agency for Innovation Promotion, formerly KTI (Project No. 44221.1 IP-LS).

### 2.5. Key Elements of the System

To handle a fully integrated supply with an orthosis along all locations within a digital process chain, the MOWA system consists of several components:

1. A gait analysis tool in which IMU sensors track the orientation of the individual leg segments, together with in-house software, making it possible to calculate the joint angles between two segments during walking.
2. Optional integration of commercially available 3D scanners allows for the precise creation of a 3D model of the lower limb (e.g., “Structure Sensor”, “Artec Eva”, “Shining 3D EinScan”) to create a 3D model of the affected body part.
3. A 3D shape software (CAD morphing software (Version 7.3)) that creates a 3D avatar of the body part; this is an integral part of the simulation app.
4. The MOWA app with a cloud-based platform as a backend, on which the necessary calculations are carried out on the basis of a comparison with corresponding data banks, and the orthosis is configured.
5. The production of the actual orthosis, consisting of preconfigured carbon parts in different designs and degrees of stiffness (subdivision into adults and children, right and left, 4 degrees of stiffness per size, 3 degrees of stiffness per sole, 3 heel heights, and 4 spring positions):
  - Ventral medial;
  - Ventral lateral;
  - Dorsal medial;
  - Dorsal lateral.

**Figure 2:**
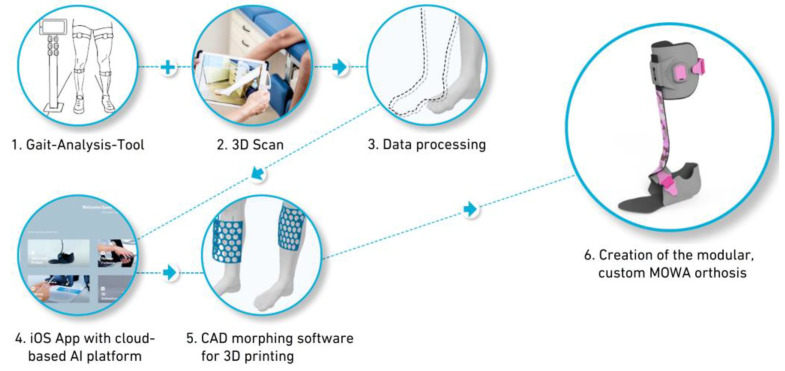
Systematic supply sequence using the MOWA system.

The stiffness of each carbon spring is determined based on forces and torque gained from gait research and simulation. In addition, 3D-printed parts, such as the individually morphed cuff and sandal (low, middle, high), as well as peripheral pieces like the clip and sensor, can be customized in terms of color and pattern according to the patient’s preference (Figure 2).

In the following sections, the individual elements of the system are explained in more detail.

### 2.6. Gait Analysis Tool and 3D Scanner

Today, gait analysis is conducted as a visual process (2D gait analysis), where the technician assesses the patient’s deficit based on the relevant parameters, such as the Modified Ashworth Scale, the Muscle Test by Janda, the range of motion of relevant joints, as well as the Amsterdam Gait Classification, and the data are entered into the MOWA app. In the future, the sensor-based MOWA gait analysis tool (MOWA GAT) will determine the accelerations occurring in the three spatial axes, as well as the rotational movements. This system is based on IMU technology (IMU = Inertial Measurement Unit) and is currently in the final phase of development [5]. It will allow the orthopedic technician to carry out an objective gait analysis regardless of location without having to rely on a complex and costly gait laboratory (3DGA). The collected data will automatically be fed into the cloud and utilized by MOWA artificial intelligence (AI) to further improve the orthosis configuration. In addition, MOWA GAT allows for the consistent documentation of changes in gait patterns over time. Consequently, the optimal orthosis adjustments can be made in a timely and cost-effective manner.

**Figure 3:**
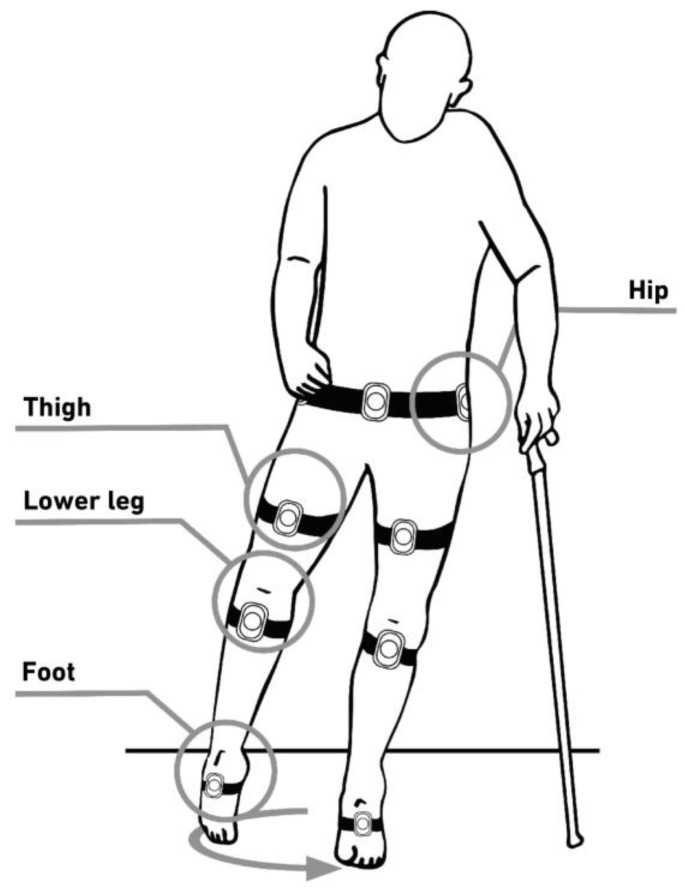
Systematic representation of where the inertial sensors for the gait analysis tool from MOWA are placed on the patient.

Furthermore, in the future, a standard 3D scanner can be used to capture the affected part of the body. The resulting data can then be uploaded into the MOWA app for automated further processing in the most common scan file formats. Currently, the technicians take manual measurements at critical positions of the patient’s leg and enter them into the MOWA app (Figure 3).

### 2.7. Three-Dimensional Shape Software

Based on the measurements entered into the MOWA app (Version 1.0), the 3D Shape Software creates an avatar of the respective leg. In the second step, the software automatically morphs the custom orthosis cuff and sandal to the patients’ anatomy, which are then ready to be 3D printed [6].

### 2.8. Cloud-Based Platform

With the cloud-based simulation app, the temporal course of joint angles can already be determined using data from two sensors fixed to the same extremity. For example, the angle of the knee joint in the gait cycle can be accurately determined by placing a sensor on the thigh and another on the lower leg. Movement restrictions are automatically detected through data comparison with authority data, and deviations in gait patterns can be detected by comparing the left leg with the right leg [7].

The determination of the optimal dimensions and form of investment of the theory is carried out automatically on the basis of classifications, such as:

- Ashworth scale (MAS) [8];
- Muscle force detection according to Janda [9];
- Amsterdam Gait Classification [10];
- GMFCS level [11];
- Activity level;
- Values from the gait analysis.

### 2.9. End Product

**Figure 4:**
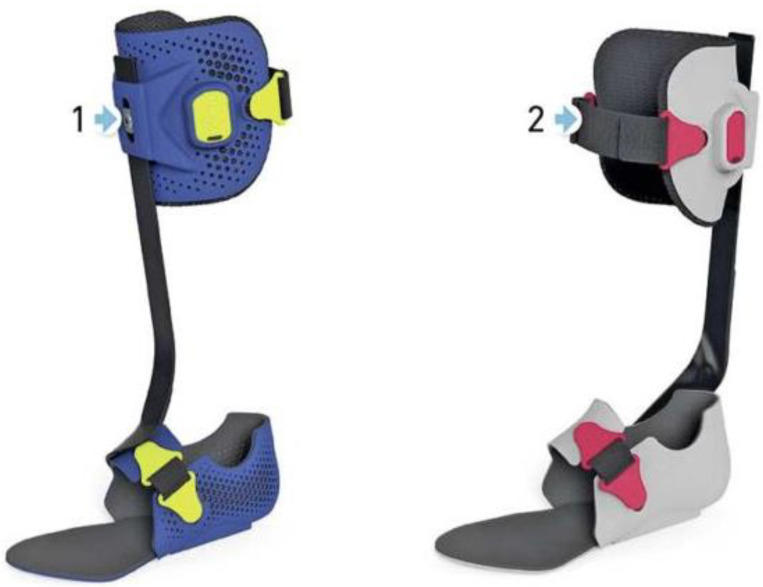
MOWA—orthosis with ventral medial (1) and with dorsal medial (2).

The end-product is a modularly constructed, bond-free custom lower leg orthosis consisting of a combination of specifically designed carbon components individually tuned according to the design and degree of stiffness in conjunction with adapted 3D-printed parts. As the only system worldwide, MOWA provides four types of spring position depending on the subject’s deficit [12]. In the case of a patient with crouch gait, for example, the simulation app recommends a ventral spring position. In a patient with genu recurvatum, a dorsal spring position is recommended, taking into account all the relevant factors (Figure 4).

With the definition of the tailored material properties of the individual elements based on scientific research and simulation, MOWA is taking a new approach in orthosis supply. The system provides seamless patient documentation and allows for the adjustment of dynamic carbon components in the event of changes, such as improvements or declines in a patient’s condition. The selection of suitable parts for each patient is performed by the MOWA AI.

### 2.10. Advantages of the MOWA System Compared to Conventional Orthoses

The new MOWA system shows various advantages over conventional orthosis fittings and will be discussed in more detail below:

### 2.11. Rapid Adaptation to Changes

An orthosis must be designed in a way that not only meets the mechanical requirements over its lifetime but also ensures the patient’s comfort while wearing it. Lightness, design, and optimal fit are particularly emphasized in the MOWA system. Thanks to its modular construction, the MOWA orthosis is an actively growing and adaptable system. Changes can be quickly and reliably detected through documentation in the MOWA app, allowing for the adjustment and exchange of components within a few minutes. This renders the production of an entirely new orthosis obsolete, saving the patient from long waiting times and enabling the technician to treat more patients in less time.

The design of the carbon spring of the orthosis is based on the patient’s collected data. Depending on the necessary support, the AI automatically selects a different configuration of spring and sole, including various heel heights. Both children’s and adult sizes are available for all parts. The carbon springs are divided into four support classes (“low”, “soft”, “flex”, “hard”). The soles are available in three stiffness levels and three heel heights: 3, 6, and 9 mm for junior sizes and 5, 10, and 15 mm for adult sizes.

### 2.12. Increase in Service Life

Custom-made orthoses manufactured based on the ‘trial-and-error principle’ often do not match the prevailing gait deficit and can often cause damage in the form of delamination and fractures to the carbon elements at an early stage. However, within the MOWA concept, the carbon components are specifically designed based on the measured forces gained through simulation. They are rigorously tested, with 2 million test cycles in the adult versions and 1 million in the junior versions, to ensure durability.

### 2.13. Intelligent Orthosis Selection

To care for patients with minimal inconvenience and within the shortest possible fitting process, it is currently necessary to try on and test different orthosis constructions several times, resulting in the use of multiple orthosis variations. However, with the MOWA concept, the need for trying on different components can be avoided. Even before the production of a MOWA orthosis, the appropriate dimensions of the orthosis and the suitable spring position are automatically determined by the AI within the MOWA app. The result is a functional compensation for the impaired muscles matching the gait deficit.

The aim is to restore a maximum symmetrical and physiological ability to walk and stand, taking into account the stance and swing phase problems. As a result, a high level of patient compliance can be achieved.

Before placing the final order, the MOWA app guides the technician to the expert mode, where the proposed configuration can be edited and changed if necessary.

## 3. Case Study

**Figure 5:**
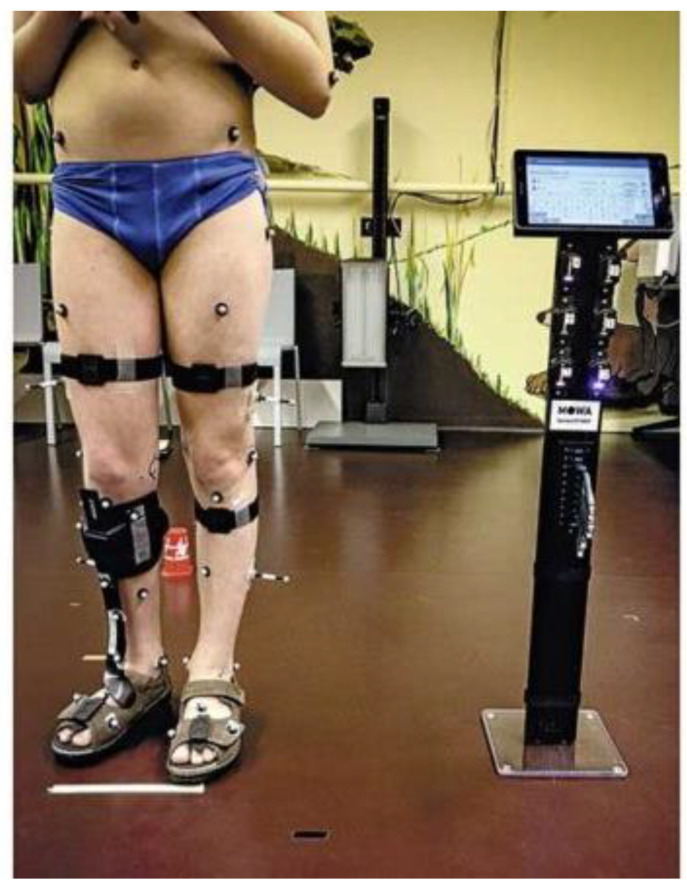
Examination in the Laboratory for Movement Analysis of the University of Basel Children’s Hospital (UKBB), Basel, with MOWA—orthosis and simultaneous gait analysis with MOWA inertial sensors.

A 13-year-old boy (GMFCS I) with unilateral spastic cerebral palsy (hemiparesis) on the right (weight 42 kg, height 151 cm) was provided with a MOWA orthosis by the authors (Figure 5). Without orthosis care, this patient experienced regular falls and intermittent foot pain. Previously, the patient had been supplied with a dynamic prefabricated carbon orthosis with a front system.

**Figure 6:**
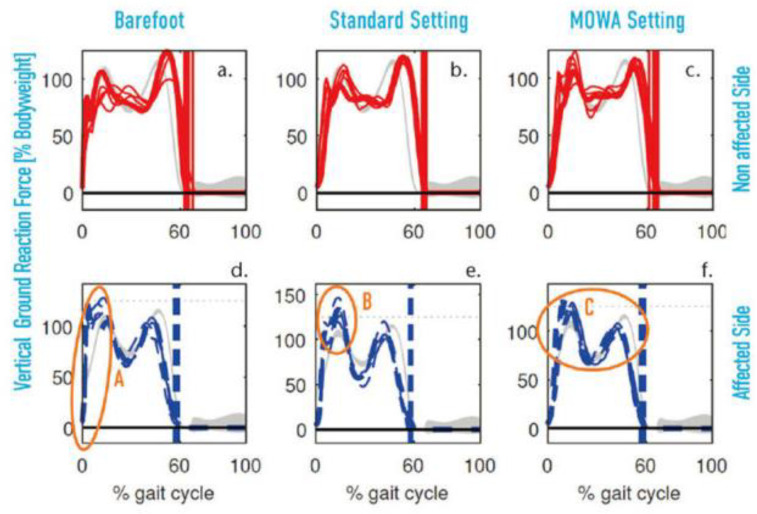
(**a**–**f**): Vertical ground reaction force for affected and non-affected side. Comparison between walking barefoot (**a**,**d**), standard orthosis (**b**,**e**), and MOWA orthosis (**c**,**f**). Vertical line at about 60%: foot leaves the ground (Foot-Off). The vertical ground reaction force shows a high load rate on the non-affected side when walking barefoot (**a**) compared to the two orthosis (**b**,**c**). However, the maximum vertical ground reaction force is higher with the standard orthosis (B) than when walking barefoot (A) and with the MOWA orthosis (C). Furthermore, the vertical ground reaction force during the stance phase with the MOWA orthosis shows a similar pattern to that of healthy subjects (C).

The 3D gait analysis resulted in the following gait pattern:

When walking barefoot, the increase in the rate of loading of the vertical ground reaction force after foot strike was greater than in healthy subjects (Figure 6a,d). This could result in higher peak loads on the musculoskeletal system. However, the vertical ground reaction force (vGRF) loading rate decreased on both sides when using the two prefabricated orthoses (Figure 6b,c,e,f). This reduction could be due to the cushioning of the shoes on the one hand and the support of the orthosis during the walking movement on the other hand.

Moreover, there was a lateral asymmetry in the size of the first maximum of the vertical ground reaction force in the dynamic prefabricated carbon orthosis (Figure 6b,e) compared to barefoot walking (Figure 6a,d) and the MOWA orthosis (Figure 6c,f). Such asymmetric gait patterns may be uncomfortable for the patient and may place additional stress on the musculoskeletal system.

On the other hand, the MOWA orthosis showed that the first peak of vGRF for both sides had a similar shape of increase and a similar maximum value (Figure 6c,f). In addition, the vertical ground reaction force pattern with the MOWA orthosis was closer to that of normal subjects than with the standard orthosis. Also, on the unaffected side, the difference between the two vertical ground reaction force maxima during the stance phase was greater when walking barefoot (Figure 6a) and with the standard brace (Figure 6b) than with the MOWA orthosis (Figure 6c).

From these results, it can be concluded that the patient using the MOWA orthosis may require less force during the push-off phase of the foot. This indicates a lower energy expenditure during walking, which could have a positive effect on gait comfort.

## 4. Limitations

The aim of this paper was to describe a new concept of a modular walking orthosis system. We aimed to outline the new chain of manufacturing, but not provide an original publication with detailed results, including full kinematics and kinetics. This will be the goal of our next project. Furthermore, we are happy to provide Supplementary Materials with four case studies, including full kinematics in the sagittal plane.

## 5. Conclusions

Thanks to the modularity of the MOWA orthosis and based on the initial findings from patient care, the authors believe that this system can better address the needs of patients by employing a scientific approach to achieve a natural and symmetrical gait pattern. MOWA can be adapted to accommodate physical changes at any time, which is particularly important for children with neurogenic gait disorders.

The aim is to further develop the MOWA supply concept through partnership-based cooperation and the use of technologies such as machine learning and clustering in a way that means that subsequent model adaptations are unnecessary.

In the near future, the MOWA orthosis will be equipped with a smart sensor to monitor patients’ activity levels and gait patterns in everyday life. This will help gain better insights into long-term changes in different diseases and support therapists in improving medically prescribed therapy programs.

Additionally, the results of the first MOWA cohort will need to be reported in detail in the future.

## 6. Patents

The MOWA concept is protected by Patent EP 2922506 B1/US 9,883,963.

## Data Availability

The data presented in this study are available on request from the corresponding author. The data are not publicly available due to patient privacy.

## Supplementary Materials

The following supporting information can be downloaded at: https://www.mdpi.com/article/10.3390/children11010030/s1, Figure S1 provides full kinematic description of four cases.
