# Supplementary material for: Novel Modular Walking Orthosis (MOWA) for Powerful Correction of Gait Deviations in Subjects with a Neurological Disease
Source: Children (Basel). 2023 Dec 26;11(1):30. doi: 10.3390/children11010030 (PMC10813927; doi:10.3390/children11010030)
Supplement: Supplementary file 1 [file children-11-00030-s001.zip › children-2767496-supplementary.pdf]

Intern:259349

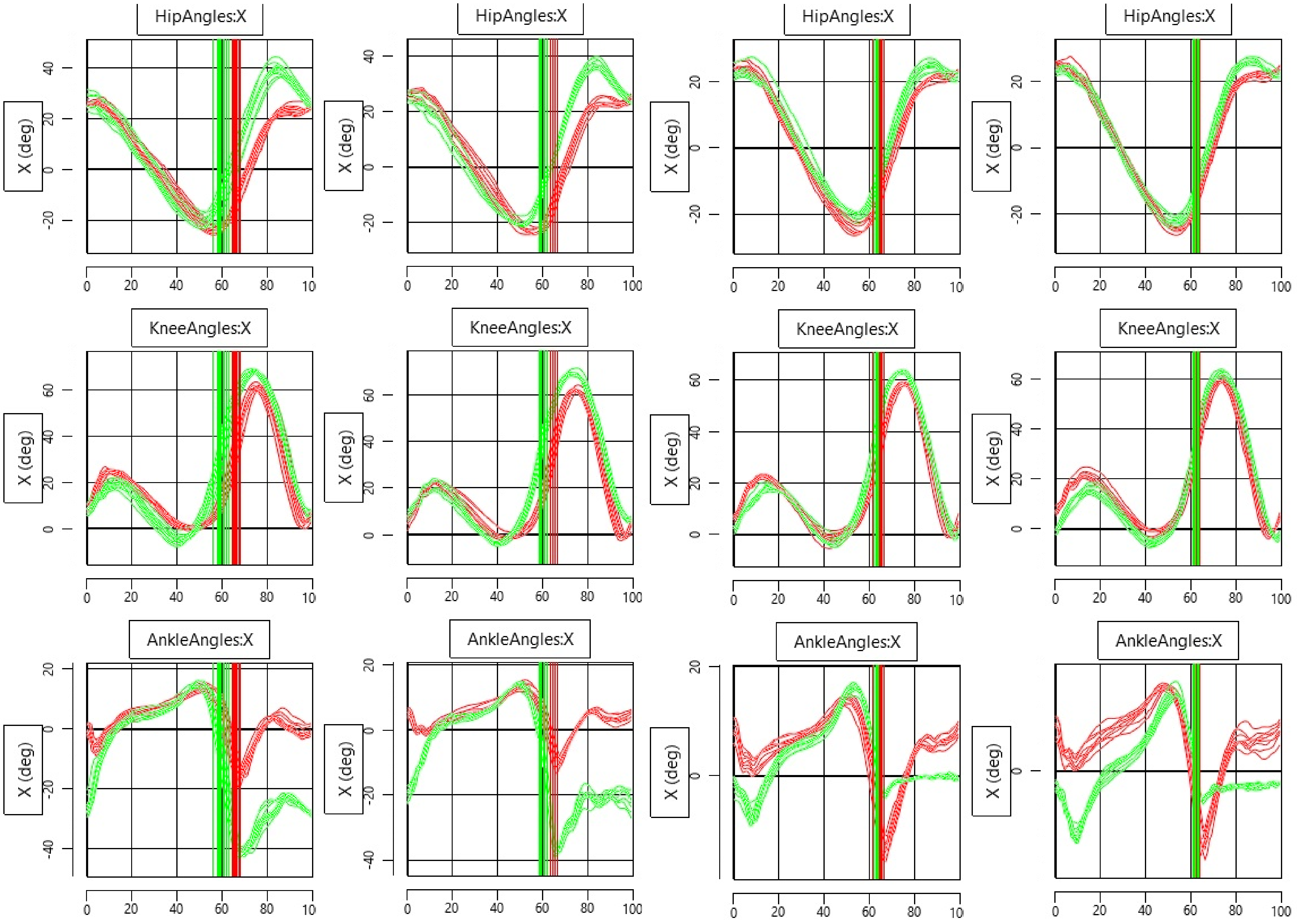

Barefoot walking
Walking speed: 1.10m/s

Walking with standard shoe
Walking speed: 1.10m/s

Walking with standard shoe
and standard AFO
Walking speed: 1.14m/s

Walking with standard shoe
and MOWA-AFO
Walking speed: 1.21m/s

Figure **S1a**: Representative kinematic data of the 12 steps sagittal angles of the hip, knee and ankle joint of a patient (m, 18y, 70.5kg, 184 cm) with a right hemiparesis wearing the first time a MOWA AFO.

The results show that the range of motion is more symmetrical in all three joints with both AFOs than barefoot or with the standard shoe. In the hip joint, the MOWA AFO achieves the same side to side symmetry in the stance phase. Whereas in the knee joint, the temporal symmetry is better with the MOWA-AFO than with the standard AFO. In the ankle joint, the push-off phase of the MOWA-AFO is 50-60% almost side symmetrical. While the remaining range of motion in the ankle joint still shows a side difference in both AFOs.

Intern: 272387

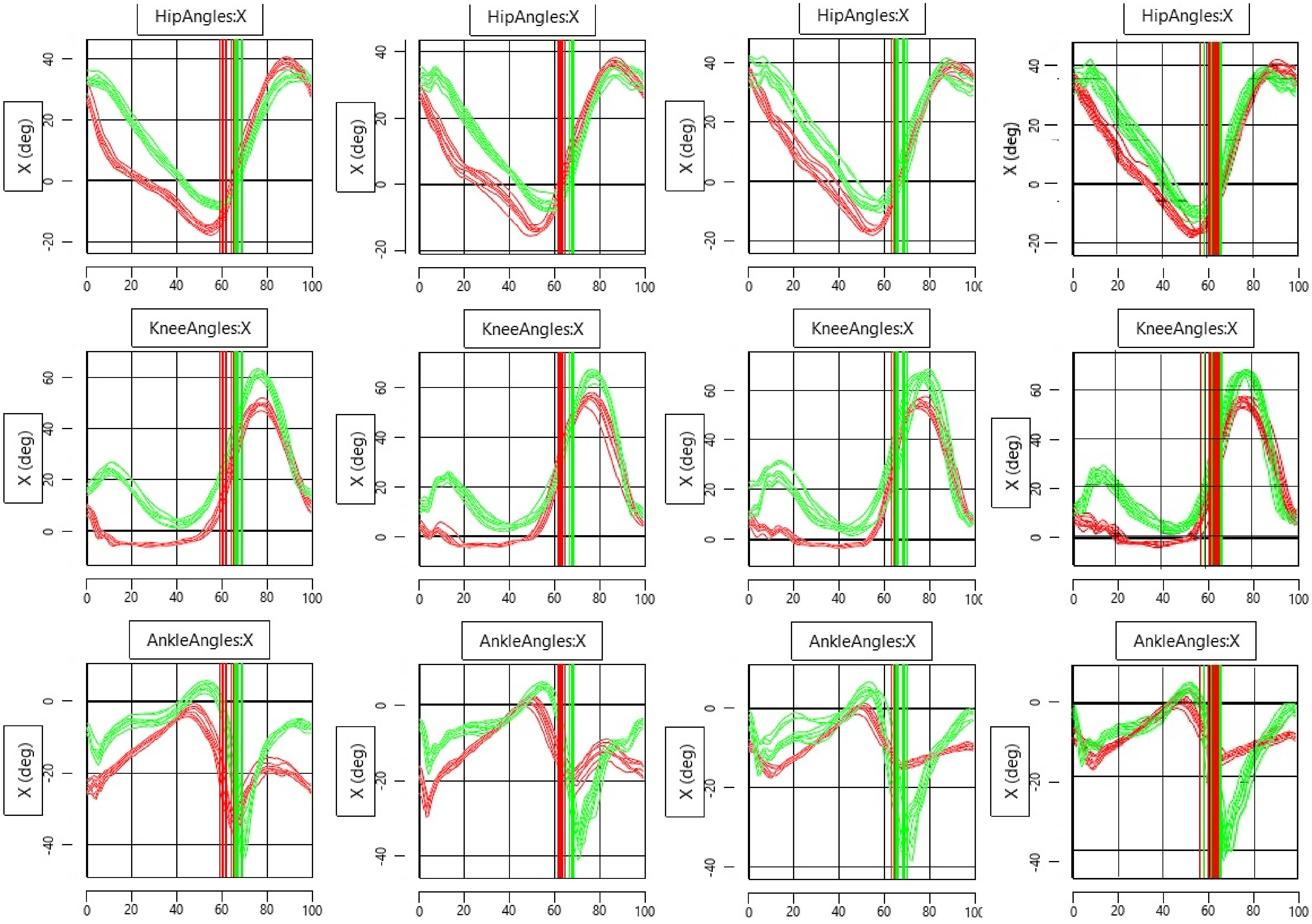

Barefoot walking
Walking speed: 1.10m/s

Walking with standard shoe
Walking speed: 1.10m/s

Walking with standard shoe and standard AFO
Walking speed: 1.14m/s

Walking with standard shoe and MOWA-AFO
Walking speed: 1.21m/s

Figure **S1b**: Representative kinematic data of the 12 steps sagittal angles of the hip, knee and ankle joint of a patient (f, 38y, 59.3kg, 163 cm) with a left hemiparesis wearing the first time a MOWA AFO.
The results show that the range of motion in the hip and ankle joint is more symmetrical with both AFOs than when walking barefoot or with the standard shoe. Whereas in the left knee joint, the side to side symmetry of both AFOs is slightly better than that of the other two conditions. Furthermore, the MOWA AFO achieves slightly better side to side symmetry in the hip and ankle joints than the standard AFO.

Intern: 705399

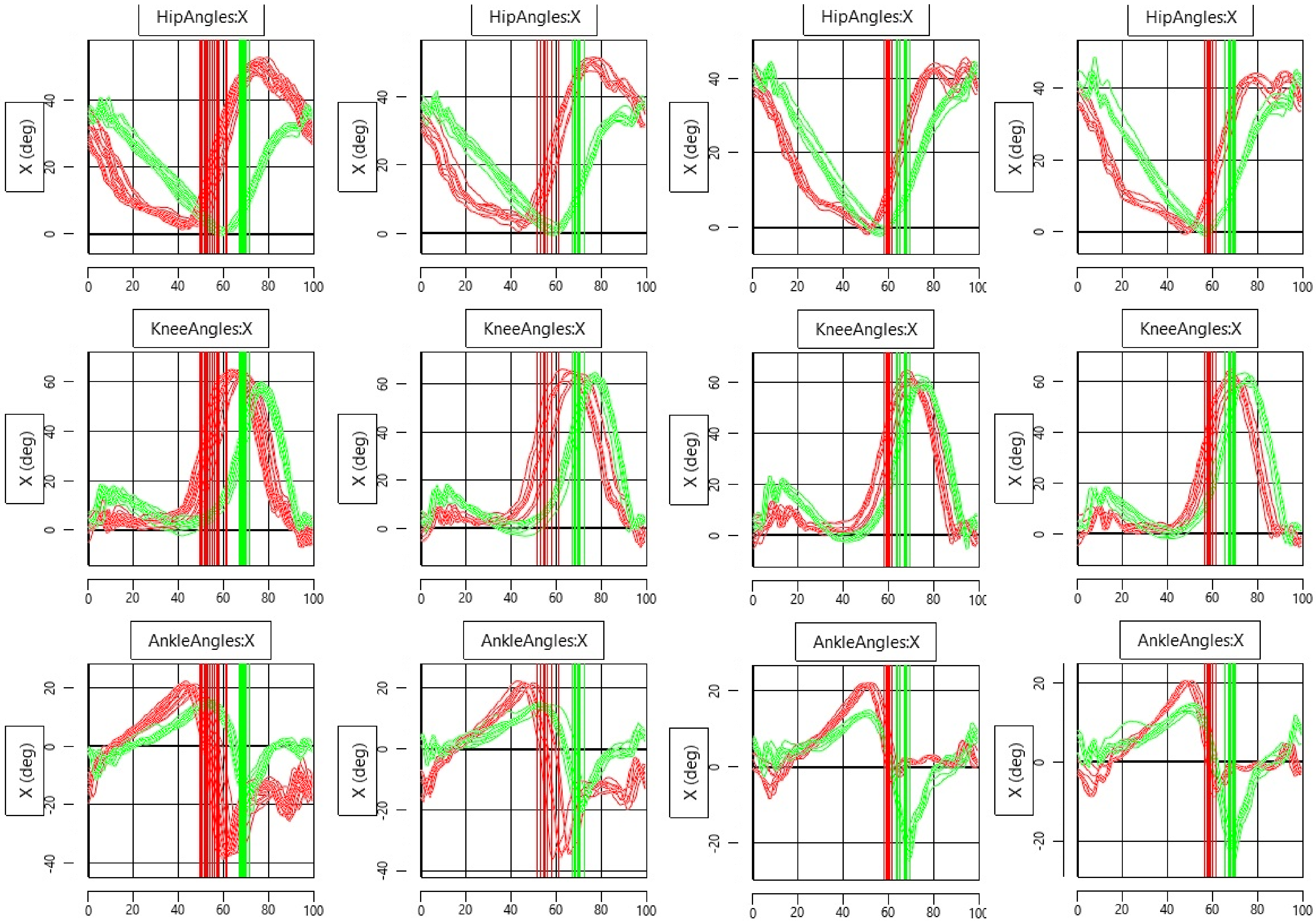

Barefoot walking
Walking speed: 0.94m/s

Walking with standard shoe
Walking speed: 1.02m/s

Walking with standard shoe and standard AFO
Walking speed: 1.28m/s

Walking with standard shoe and MOWA-AFO
Walking speed: 1.25m/s

Figure **S1c**: Representative kinematic data of the 12 steps sagittal angles of the hip, knee and ankle joint of a patient (m, 15y, 54.1 kg, 167cm) with a left hemiparesis wearing the first time a MOWA AFO.

The results show that the range of motion is more symmetrical in all three joints with both AFOs than barefoot or with the standard shoe. In the hip joint, the Standard-AFO has a slight higher side to side symmetry than the MOWA-AFO. Whereas in the knee joint, the MOWA-AFO is better than with the standard AFO. The side to side symmetry in the ankle joint is better in the early stance phase with the standard AFO, but better in the push-off phase with the MOWA AFO.

Intern: 944711

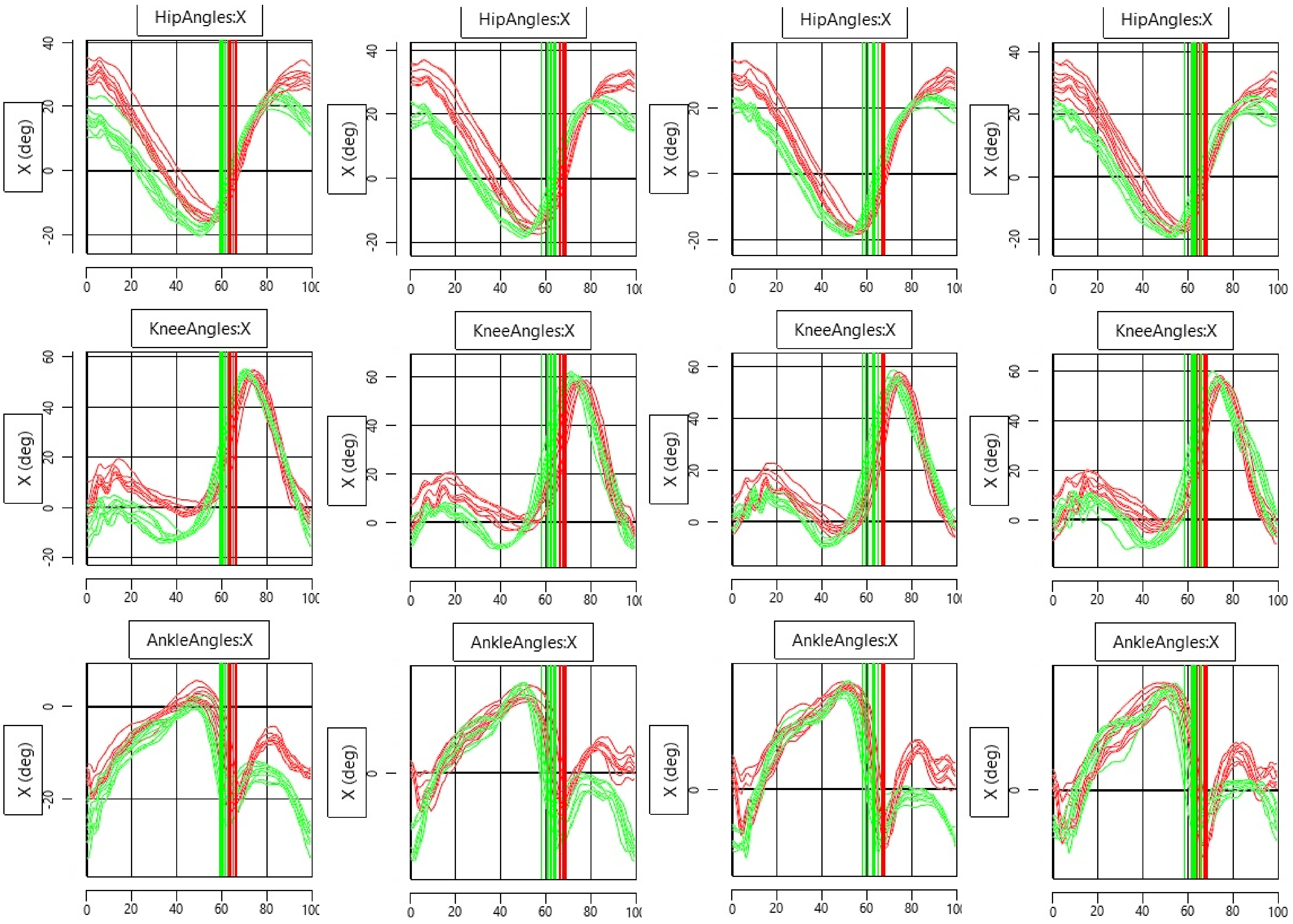

Barefoot walking
Walking speed: 0.92 m/s

Walking with standard shoe
Walking speed: 0.98m/s

Walking with standard shoe
and standard AFO
Walking speed: 1.01 m/s

Walking with standard shoe
and MOWA-AFO
Walking speed: 1.10m/s

Figure **S1d**: Representative kinematic data of the 12 steps sagittal angles of the hip, knee and ankle joint of a patient (m, 22 y, 89.6kg, 180cm) with a right hemiparesis wearing the first time a MOWA AFO.

The results show that the range of motion is more symmetrical in all three joints with both AFOs than barefoot or with the standard shoe. In the hip and ankle joints, the MOWA AFO achieves slightly better side to side symmetry than the standard AFO. In the knee joint, the difference is nearly invisible.
